# Association of Arterial Velocity Pulse Index and Arterial Pressure–Volume Index with Central Arterial Stiffness and Cardiac Function in the Japanese Population

**DOI:** 10.3390/jcm15041317

**Published:** 2026-02-07

**Authors:** Hiroto Hommo, Takuya Sugawara, Hikaru Ueno, Honoka Kawashima, Kotaro Uchida, Shintaro Minegishi, Lin Chen, Rie Sasaki-Nakashima, Tabito Kino, Kentaro Arakawa, Michiko Sugiyama, Noriyuki Kawaura, Koichi Tamura, Kiyoshi Hibi, Tomoaki Ishigami

**Affiliations:** 1 Department of Cardiology, Yokohama City University, Yokohama 236-0004, Japan; e213075c@yokohama-cu.ac.jp (H.H.); takuya09061911617@hotmail.co.jp (T.S.); e213016g@yokohama-cu.ac.jp (H.U.); e213028e@yokohama-cu.ac.jp (H.K.); swinging_jazz_life@yahoo.co.jp (K.U.); minegishi.shi.fb@yokohama-cu.ac.jp (S.M.); lin.chen@yokohama-cu.ac.jp (L.C.); t136036f@yokohama-cu.ac.jp (R.S.-N.); kino-tabito@umin.ac.jp (T.K.); hiroking@gamma.ocn.ne.jp (K.A.); vn_nv2525smile@yahoo.co.jp (M.S.); nkawaura@yokohama-cu.ac.jp (N.K.); hibikiyo@yokohama-cu.ac.jp (K.H.); 2 Department of Cardiology, Sir Run Run Hospital, Nanjing Medical University, Long Mian Avenue 109 Jiangning, Nanjing 210029, China; 3 Department of Cardio-Renal Medicine, Yokohama City University, Yokohama 236-0004, Japan; tamukou@yokohama-cu.ac.jp

**Keywords:** arterial stiffness, pulse wave analysis, AVI, API, echocardiography, central hemodynamics

## Abstract

**Background:** The Arterial Velocity Pulse Index (AVI) and Arterial Pressure–Volume Index (API) are novel non-invasive indices of arterial stiffness derived from cuff-oscillometric measurements. Previous studies have shown that elevated AVI and API are associated with the severity of coronary artery disease and the ability to predict future cardiovascular events. However, the hemodynamic and echocardiographic characteristics of patients with concomitantly high AVI and API remain unclear. **Methods:** We retrospectively analyzed 112 consecutive cardiovascular outpatients (mean age 69.1 ± 12.2 years, 64.3% male) seen between January and April 2019 at Yokohama City University Hospital. The AVI and API were measured using a multifunctional sphygmomanometer (PASESA AVE1500, Shisei Datum, Japan) and averaged over a maximum of three measurements. Patients were classified into four groups according to previously established cutoff values (AVI ≥ 27, API ≥ 32). Central arterial pulse wave parameters were assessed using SphygmoCor XCEL (AtCor Medical, Sydney, Australia), and echocardiographic parameters were obtained according to standard protocols. Intergroup differences were analyzed using the Kruskal–Wallis test with Steel–Dwass post hoc comparisons. **Results:** Compared with the low-risk group (low AVI/low API), the high-risk group (high AVI/high API) had significantly higher brachial systolic BP (139.2 [132.8–149] vs. 128 [120–136.7] mmHg, *p* = 0.0011), central systolic BP (127.5 [122.3–139] vs. 117.7 [110.3–123.7] mmHg, *p* = 0.0018), and central pulse pressure (56.2 [51.4–60.3] vs. 37.7 [32–43] mmHg, *p* < 0.001). The forward and reflected wave amplitudes were significantly greater, with prolonged ejection duration and aortic T2 time. The Buckberg subendocardial viability ratio was significantly lower in the high-risk group (129.5 [119.7–145.2] vs. 148.3 [130–168.3], *p* = 0.040). Echocardiography revealed reduced e′ velocity (5 [4.1–5.8] vs. 6.7 [5.2–8] cm/s, *p* = 0.035) and increased E/e′ (13.2 [11.1–15.1] vs. 9.7 [7.9–11.3], *p* = 0.026) in the high-risk group, suggesting the presence of impaired diastolic function without reduced LVEF. **Conclusions:** Patients with high AVI and API exhibited greater central and peripheral arterial stiffness, higher systolic and pulse pressures, and impaired diastolic function compared with those with low values. These findings support the use of a cardiovascular pathophysiological model in which elevated AVI/API identify individuals at increased risk of progression to heart failure and ischemic heart disease.

## 1. Introduction

Arterial stiffness is a recognized marker of vascular aging and an independent predictor of cardiovascular morbidity and mortality [1,2,3,4]. The development of reliable, non-invasive, and easily obtainable arterial stiffness indices is therefore of significant clinical interest.

In recent years, oscillometric techniques have enabled the assessment of arterial stiffness using simple cuff-based blood pressure measurements [5,6]. Using such an approach, the Arterial Velocity Pulse Index (AVI) and the Arterial Pressure–Volume Index (API) can be obtained with the PASESA AVE1500 system (Shisei Datum, Tokyo, Japan) [5]. The AVI reflects the characteristics of central arterial stiffness by analyzing brachial pulse waveforms recorded during suprasystolic cuff inflation, whereas the API provides an estimate of peripheral arterial compliance based on the pressure–volume relationship observed during cuff deflation.

We, along with other researchers, have previously demonstrated that both the AVI and API are correlated with the severity and complexity of coronary artery disease, and that threshold values of 27 (AVI) and 32 (API) predict cardiovascular events [1,7,8,9]. However, the cardiovascular phenotype of patients with simultaneously high AVI and API—representing both central and peripheral stiffness—remains insufficiently characterized. To date, no study has comprehensively evaluated central pulse wave characteristics and echocardiographic findings across the four AVI/API-defined categories. Therefore, whether concomitantly elevated AVI and API reflect an adverse cardiovascular phenotype remains unknown.

This study aimed to classify patients into four groups according to AVI and API cutoff values and compare central pulse wave parameters and echocardiographic findings between these groups. We hypothesized that patients with concomitantly elevated AVI and API would exhibit more adverse hemodynamic profiles and evidence of subclinical myocardial dysfunction.

## 2. Materials and Methods

### 2.1. Study Population

We retrospectively reviewed 112 consecutive outpatients who attended the Department of Cardiology at Yokohama City University Hospital between January and April 2019. The inclusion criteria were age ≥ 20 years and the availability of both AVI and API measurements, along with central pulse wave analysis and echocardiographic data. Patients with arrhythmias that interfered with the pulse wave measurements, severe valvular disease, or acute cardiovascular events within three months were excluded from this study. Because patient inclusion required successful assessment with both PASESA and SphygmoCor XCEL, the study population represents a selected cohort, and the analysis was exploratory in nature.

### 2.2. Measurement of AVI and API

The oscillometric assessment of arterial stiffness was performed using the PASESA AVE1500 system (Shisei-Datum, Tokyo, Japan). This device acquires brachial pulse waveforms during suprasystolic cuff inflation to derive the Arterial Velocity Pulse Index (AVI) and analyzes the pressure–volume relationship obtained during cuff deflation to calculate the Arterial Pressure–Volume Index (API). Measurements were obtained in the seated position after at least 5 min of rest in the outpatient clinic. The average of a maximum of three measurements was used for the analysis [7,8].

### 2.3. Group Classification

Patients were classified into four groups:

**Group I:** Low AVI (<27)/Low API (<32).

**Group II:** Low AVI (<27)/High API (≥32).

**Group III:** High AVI (≥27)/Low API (<32).

**Group IV:** High AVI (≥27)/High API (≥32).

### 2.4. Central Pulse Wave Analysis

Central hemodynamic parameters, including central systolic and diastolic BP, pulse pressure, augmentation pressure, augmentation index (AIx), ejection duration, aortic T2, forward and reflected wave amplitudes, and the Buckberg subendocardial viability ratio (SEVR), were assessed using SphygmoCor XCEL (AtCor Medical, Sydney, Australia). The average of a maximum of three measurements was used for the analysis.

### 2.5. Echocardiography

Standard transthoracic echocardiography was performed in accordance with the American Society of Echocardiography guidelines [10]. Left ventricular dimensions, ejection fraction (EF), diastolic filling parameters (E/A ratio, septal e′, and E/e′), and left atrial diameter were measured. All echocardiographic measurements were performed by experienced, certified cardiac sonographers under the supervision of cardiologists, as part of routine clinical practice at a university hospital, in accordance with the established guidelines. Although formal intra- and inter-observer variability analyses were not conducted for research purposes in this retrospective study, the measurements were obtained using standardized protocols routinely applied in daily clinical care.

### 2.6. Statistical Analysis

Continuous variables are presented as mean ± standard deviation (SD) or median [interquartile range], and categorical variables are presented as number and percentage. Intergroup comparisons of continuous variables were made using Welch’s one-way analysis of variance (ANOVA) followed by Bonferroni’s multiple comparison test, or the Kruskal–Wallis test with the Steel–Dwass test. Omnibus tests and pairwise comparisons of categorical variables were performed using the chi-square test or Fisher’s exact test. Pairwise comparisons were corrected by Bonferroni correction. A two-tailed *p*-value < 0.05 was considered statistically significant. All statistical analyses were performed with EZR (version 1.68; Jichi Medical University, Tochigi, Japan), which is a graphical user interface for R (The R Foundation for Statistical Computing, Vienna, Austria) [11].

## 3. Results

**Figure 1:**
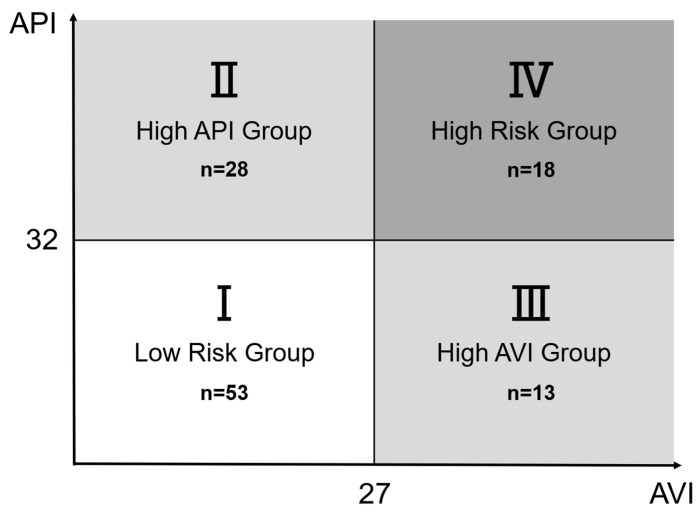
Group definition by AVI and API.

A total of 112 patients were analyzed and stratified into four groups according to the AVI/API cutoff values (Group I, n = 53; Group II, n = 28; Group III, n = 13; Group IV, n = 18, Figure 1). The baseline characteristics are shown in Table 1. The patients in Groups II–IV tended to be older than those in Group I, with the highest mean age observed in Group IV. The prevalence of diabetes mellitus increased progressively from Group I to Group IV, and Group II showed the highest proportion of antidiabetic use and median HbA1c value. The use of hypolipidemic agents also showed a stepwise increase with the elevation of AVI/API values. Other comorbidities and medication use were generally comparable among the groups.

The subjects (n = 112) were classified into four groups according to their API and AVI values. Group I included subjects with standard API (<32) and AVI (<27). Group II included those with elevated API only (API ≥ 32 and AVI < 27). Group III included those with elevated AVI only (API < 32 and AVI ≥ 27). Group IV included those with both elevated API and AVI (API ≥ 32 and AVI ≥ 27). In total, 53 subjects were assigned to Group I, 28 to Group II, 13 to Group III, and 18 to Group IV.

Hemodynamic parameters

**Figure 2:**
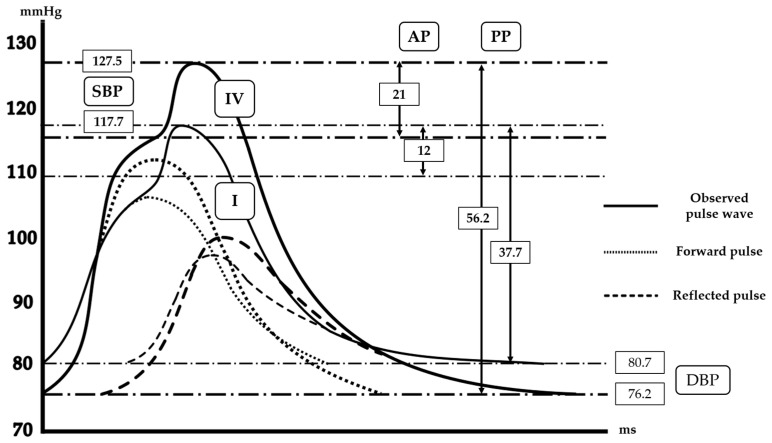
Schematic presentation of the differences in central pulse wave forms between the subjects of Groups I and IV. Solid lines indicate the observed central pulse waveforms, dotted lines indicate the forward pressure wave, and dashed lines indicate the reflected pressure wave. Group I represents subjects with low AVI and API, whereas Group IV represents subjects with high AVI and API. SBP indicates systolic blood pressure; DBP, diastolic blood pressure; PP, pulse pressure; AP, augmentation pressure. Values shown are median values.

Hemodynamic data are summarized in Table 2 and Figure 2. Brachial systolic blood pressure showed a gradual increase across the groups, with Group IV demonstrating the highest values (139.2 [132.8–149] mmHg), significantly higher than those in Group I (128 [120–136.7] mmHg, *p* = 0.0011). A similar stepwise pattern was observed in terms of central systolic blood pressure and central pulse pressure, both of which rose progressively from Group I through to Group IV.

Indices reflecting wave reflection also demonstrated clear gradients. P1 height, forward pulse height, and reflected pulse height increased consistently across the four groups, with marked differences between Group IV and Group I (all *p* < 0.001). Augmentation pressure and ejection duration similarly exhibited progressive elevations from lower to higher AVI/API categories. Although the augmentation index and reflection magnitude did not differ significantly between the groups, those of Group IV were elevated compared with those of the other groups.

Echocardiographic findings

The echocardiographic data are presented in Table 3. Left ventricular size, left atrial diameter, and cardiac output were generally comparable among the four groups. The ejection fraction of Group II was significantly higher than that of Groups I and IV, but it was not significant between Group I and Group IV. However, the indices of diastolic function demonstrated stepwise deterioration in association with higher AVI/API values. Specifically, the e′ velocity decreased gradually from Group I (6.7 [5.2–8] cm/s) to Group IV (5 [4.1–5.8] cm/s) (*p* = 0.035 for Group IV vs. Group I), while E/e′ increased in a parallel manner (13.2 [11.1–15.1] to 9.7 [7.9–11.3], *p* = 0.026 for Group IV vs. Group I). These findings suggest that progressive vascular stiffening, as captured by the AVI/API, is accompanied by worsening left ventricular relaxation and elevated filling pressures.

## 4. Discussion

In this exploratory, retrospective cross-sectional study, we evaluated the prognostic significance of arterial stiffness indices, the Arterial Velocity Pulse Index (AVI), and the Arterial Pressure–Volume Index (API). Our principal findings were as follows: Firstly, when stratifying patients into four groups according to AVI/API cutoff values, which we proposed previously, the high-risk group (Group IV) demonstrated significant differences in several hemodynamic parameters compared with the low-risk group (Group I). In particular, central blood pressure (central SBP, augmentation pressure), indices of pulse wave reflection (P1 height, reflected pulse height, forward pulse height), and ejection duration were markedly elevated in Group IV. Secondly, the echocardiographic assessment revealed evidence of impaired diastolic function, including reduced e′ and increased E/e′, in the high-AVI/API group. These results suggest that patients with elevated AVI/API not only exhibit increased arterial stiffness but also subclinical left ventricular (LV) diastolic dysfunction.

From a clinical perspective, these findings are notable. The AVI and API are conventionally regarded as surrogate indices of arterial stiffness [7,8,9,12], yet our results imply that their significance extends beyond vascular mechanics. The observed associations with echocardiographic parameters indicate that the AVI/API may reflect LV filling pressures and diastolic function, thereby serving as markers that bridge vascular and cardiac dysfunction. The simultaneous elevation of forward and reflected wave amplitudes in Group IV suggests increased impedance mismatch and accelerated wave return, which are known to contribute to higher central systolic load and impaired relaxation. Given that AVI/API assessment is simple and non-invasive, these indices may offer a practical tool for risk stratification in daily clinical practice, particularly for identifying individuals at risk of adverse cardiovascular outcomes.

The present findings suggest that concomitantly elevated AVI and API values reflect a pathophysiological state characterized by increased arterial stiffness at both central and peripheral levels, which may adversely affect left ventricular diastolic function. Elevated AVI has been shown to represent increased central arterial stiffness and enhanced wave reflection, leading to higher central systolic pressure and augmented late systolic load on the left ventricle [13,14]. This increase in pulsatile afterload may impair active myocardial relaxation and contribute to diastolic dysfunction, even in the absence of overt systolic impairment [15,16,17].

In parallel, elevated API reflects reduced peripheral arterial compliance, which may further exacerbate abnormalities in ventricular–vascular coupling. Increased peripheral stiffness can alter the timing and magnitude of reflected pressure waves, reduce diastolic pressure augmentation, and impair coronary perfusion during diastole [18,19]. Such hemodynamic alterations may be particularly relevant to subclinical myocardial dysfunction, as reflected by impaired diastolic parameters such as reduced e′ velocity and elevated E/e′ ratio.

Therefore, the combined elevation of the AVI and API may represent a hemodynamically unfavorable phenotype in which both central pulsatile load and peripheral arterial compliance are compromised [20,21]. This condition may accelerate age-related ventricular–vascular stiffening and promote the development of diastolic dysfunction, a key pathophysiological feature of heart failure with preserved ejection fraction (HFpEF). Our findings support the concept that simple cuff-based arterial stiffness indices may capture clinically meaningful vascular–cardiac interactions and help identify individuals at risk of early myocardial dysfunction before the onset of overt heart failure.

Our findings are consistent with prior studies that have validated the AVI and API in comparison with carotid–femoral pulse wave velocity (ba-PWV) [3,22], one of the standard measures of arterial stiffness. Several reports have demonstrated that the AVI and API correlate with central blood pressure and augmentation indices, thereby supporting their validity as vascular biomarkers [7,8,9,23]. However, the present study extends existing knowledge by demonstrating a significant association between the AVI/API and echocardiographic indices such as e′ and E/e′. This novel observation suggests that there is a potential mechanistic link between increased arterial stiffness and LV diastolic impairment, warranting further exploration in future investigations. Future studies incorporating complementary diagnostic modalities, such as myocardial strain imaging, circulating biomarkers of myocardial stress, or cardiac magnetic resonance imaging, may further substantiate the mechanistic link between arterial stiffness and subclinical myocardial dysfunction.

This study has several limitations that should be acknowledged. Firstly, this was a single-center, retrospective study with a relatively small sample size and a short enrollment period, which may limit the generalizability of the findings. Secondly, patient inclusion was restricted to those who underwent both oscillometric arterial stiffness assessment (AVI/API using PASESA) and central hemodynamic evaluation using SphygmoCor XCEL, resulting in a selected study population and an exploratory study design. Thirdly, the number of clinical events was limited, precluding definitive conclusions regarding prognostic outcomes. In addition, although the sex distribution was documented, the limited sample size did not allow for robust sex-specific analyses. Because formal multivariable regression analyses were not performed, we cannot exclude the possibility that age-related differences may have contributed to the observed intergroup differences, particularly between the low-risk and high-risk AVI/API categories. Future multicenter prospective cohort studies and interventional trials with larger populations are warranted to validate the prognostic significance of AVI/API cutoff values and clarify their role in cardiovascular risk stratification.

## 5. Conclusions

In conclusion, the present study demonstrates that concomitantly elevated AVI and API values are associated with adverse central hemodynamics and impaired left ventricular diastolic function in a Japanese outpatient population. These findings suggest that the combined assessment of central and peripheral arterial stiffness using simple cuff-based oscillometric indices may provide clinically meaningful insights into subclinical myocardial dysfunction. Given their non-invasive nature and ease of use in routine clinical practice, the AVI and API may serve as practical tools for early cardiovascular risk stratification, particularly in patients at risk of diastolic dysfunction and heart failure with preserved ejection fraction. Further large-scale, prospective studies are warranted to confirm these observations and determine whether targeted interventions based on AVI/API profiles can improve clinical outcomes.

## Figures and Tables

**Table 1:** Baseline characteristics.

	Overall	Group I	Group II	Group III	Group IV
	(n = 112)	(n = 53)	(n = 28)	(n = 13)	(n = 18)
Gender (%) *	72 (64.3%)	42 (79.2%)	14 (50.0%)	5 (38.5%)	11 (61.1%)
Age *	69.1 ± 12.2	62.8 ± 13.0	74.6 ± 8.5	73.8 ± 9.0	75.3 ± 8.0
BMI (kg/m^2^)	23.5 [21.8–25.8]	24.4 [22.1–26.6]	23.3 [22.1–25.1]	21.9 [20.0–25.6]	23.4 [21.8–24.1]
Smoking (%)	16 (15.4%) (n = 104)	9 (18.8%) (n = 48)	3 (11.5%) (n = 26)	1 (7.7%) (n = 13)	3 (17.6%) (n = 17)
HT (%)	68 (60.7%)	28 (52.8%)	20 (71.4%)	8 (61.5%)	12 (66.7%)
DM (%) *	30 (26.8%)	7 (13.2%)	12 (42.9%)	2 (15.4%)	9 (50.0%)
HL (%)	31 (27.7%)	12 (22.6%)	8 (28.6%)	3 (23.1%)	8 (44.4%)
CKD (%)	27 (24.1%)	11 (20.8%)	9 (32.1%)	2 (15.4%)	5 (27.8%)
CAD (%)	32 (28.6%)	14 (26.4%)	7 (25.0%)	5 (38.5%)	6 (33.3%)
PAD (%)	0 (0.0%)	0 (0.0%)	0 (0.0%)	0 (0.0%)	0 (0.0%)
CHF (%)	6 (5.4%)	3 (5.7%)	1 (3.6%)	2 (15.4%)	0 (0.0%)
VHD (%)	12 (10.7%)	6 (11.3%)	1 (3.6%)	1 (7.7%)	4 (22.2%)
Af (%)	14 (12.5%)	7 (13.2%)	5 (17.9%)	1 (7.7%)	1 (5.6%)
Hemodialysis (%)	2 (1.8%)	1 (1.9%)	0 (0.0%)	0 (0.0%)	1 (5.6%)
Medication					
Antihypertensive (%)	91 (81.3%)	44 (83.0%)	23 (82.1%)	10 (76.9%)	14 (77.8%)
Hypolipidemic Agent (%) *	52 (46.4%)	17 (32.1%)	17 (60.7%)	7 (53.8%)	11 (61.1%)
Antidiabetic Agent (%) *	19 (17.0%)	3 (5.7%)	9 (32.1%)	2 (15.4%)	5 (27.8%)
Laboratory data					
BUN (mg/dL)	17 [14–19] (n = 98)	16 [13–19] (n = 47)	17 [15–20] (n = 23)	15 [14.8–17.3] (n = 12)	17.5 [15.8–21] (n = 16)
Creatinine (mg/dL)	0.87 [0.76–1.07] (n = 107)	0.89 [0.81–1.03] (n = 50)	0.87 [0.69–1.09] (n = 27)	0.82 [0.72–0.88] (n = 12)	0.89 [0.77–1.14] (n = 18)
eGFR (mL/min/1.73m^2^)	61.8 [51.0–69.8] (n = 107)	64.9 [52.8–70.9] (n = 50)	60.3 [47.6–66.7] (n = 27)	51.3 [50.4–71.3] (n = 12)	59.9 [45.6–64.4] (n = 18)
Uric Acid (mg/dL)	5.5 [4.8–6.4] (n = 60)	5.4 [4.9–6] (n = 23)	5.5 [3.9–6.7] (n = 23)	5.6 [5.1–5.6] (n = 6)	5.3 [4.7–6.1] (n = 8)
Na (mmol/L)	142 [141–144] (n = 103)	142 [141–144] (n = 46)	142 [141–143.5] (n = 27)	143 [140.5–144] (n = 12)	142 [142–143] (n = 18)
K (mmol/L)	4.3 [4.1–4.5] (n = 103)	4.3 [4.1–4.6] (n = 46)	4.2 [4–4.4] (n = 27)	4.3 [3.8–4.6] (n = 12)	4.4 [4.2–4.5] (n = 18)
Cl (mmol/L)	105 [103–106] (n = 101)	105 [103–105] (n = 45)	105 [103.3–107] (n = 26)	105.5 [102–106.3] (n = 12)	105 [105–106] (n = 18)
HbA1c (%) *	6.1 [5.7–6.5] (n = 64)	5.9 [5.7–6.3] (n = 31)	6.5 [6.2–7.0] (n = 16)	5.8 [5.7–5.9] (n = 5)	6.2 [5.8–6.3] (n = 12)
BNP (pg/mL)	31.7 [13.7–74.1] (n = 64)	32.3 [13.9–101.1] (n = 32)	34.6 [16.3–54.6] (n = 14)	41.3 [19.3–85.7] (n = 7)	21.7 [16.0–40.4] (n = 11)
LDL-C (mg/dL)	101 [79–125.4] (n = 87)	102 [83.3–129] (n = 40)	89 [78.5–122.3] (n = 24)	97 [85.5–132.6] (n = 8)	116 [80–121.5] (n = 15)
HDL-C (mg/dL)	60 [49–73.8] (n = 86)	62 [46–75] (n = 39)	60.5 [50.5–69.5] (n = 24)	66.5 [56–86.3] (n = 8)	58 [48–61] (n = 15)
TC (mg/dL)	193 [164–219.8] (n = 50)	189 [167–222] (n = 25)	193 [158–207] (n = 15)	226 [196.3–252.3] (n = 4)	189 [157.3–197.5] (n = 6)
TG (mg/dL)	135 [92.5–193] (n = 87)	131 [92–198] (n = 41)	137 [89.5–249] (n = 23)	138.5 [117.8–147.3] (n = 8)	142 [123.5–153.5] (n = 15)
AVI	21.2 [18.3–28.4]	19.7 [16–22]	19.7 [18.2–22]	29.3 [28.7–30.3]	32.8 [31.4–35.6]
API	29.5 [24.7–34.2]	24.3 [21–27.3]	37.7 [33.7–43.1]	29.3 [27.7–31]	34.7 [33.1–38.7]

Data are presented as mean ± standard deviation for parametric data, median [interquartile range] for nonparametric data, and n (%) for categorical data. The data in the gender tables indicate the number of males. For items with missing values, the number of subjects whose values were obtained are indicated in the tables. Some low-density lipoprotein cholesterol (LDL-C) and high-density lipoprotein cholesterol (HDL-C) values were calculated using the Friedewald Equation. Asterisk marks were added to items which showed significant differences among the four groups. BMI, body mass index; HT, hypertension; DM, diabetes mellitus; HL, hyperlipidemia; CKD, chronic kidney disease; CAD, coronary artery disease; PAD, peripheral arterial disease; CHF, chronic heart failure; VHD, valvular heart disease; Af, atrial fibrillation; BUN, blood urea nitrogen; eGFR, estimated glomerular filtration rate; BNP, brain natriuretic peptide; LDL-C, low-density lipoprotein cholesterol; HDL-C, high-density lipoprotein cholesterol; TC, total cholesterol; TG, triglyceride; AVI, Arterial Velocity Pulse Index; API, Arterial Pressure–Volume Index.

**Table 2:** Central hemodynamic parameters: comparison of pulse wave indices between Group I and Group IV.

	Group I	Group IV	Group I vs. Group IV
	(n = 53)	(n = 18)	
**Brachial SBP (mmHg)**	**128 [120**–**136.7]**	**139.2 [132.8**–**149]**	***p*** **= 0.0011**
Brachial DBP (mmHg) *	79.3 [71.3–87]	75.7 [64.6–80.6]	*p* = 0.37
**Central SBP (mmHg)**	**117.7 [110.3**–**123.7]**	**127.5 [122.3**–**139]**	***p*** **= 0.0018**
Central DBP (mmHg) *	80.7 [71.7–87]	76.2 [65.3–80.8]	*p* = 0.39
**Central PP (mmHg)**	**37.7 [32**–**43]**	**56.2 [51.4**–**60.3]**	***p*** **< 0.001**
Central MP (mmHg)	96 [89.7–103.3]	96 [88.3–104.9]	*p* = 0.98
MAP Systole (mmHg)	105.7 [99.3–114]	112.8 [105.8–121.8]	*p* = 0.077
MAP Diastole (mmHg) *	90.7 [82.3–98]	87.3 [79.1–91.7]	*p* = 0.83
Heart Rate (bpm)	75.7 [62.3–81.3]	70.8 [60.3–74.5]	*p* = 0.46
**Central AP (mmHg)**	**12 [8.7**–**16.3]**	**21 [17.1**–**25.3]**	***p*** **< 0.001**
Central AIx (%)	32.3 [22.3–40.3]	39.5 [32.8–45.1]	*p* = 0.14
**Ejection Duration (ms)**	**294.3 [279.7**–**314.3]**	**324.2 [310.5**–**349.5]**	***p*** **= 0.0063**
Ejection Duration (%)	36.3 [33.3–39.3]	37.3 [33.5–39.6]	*p* = 0.97
**Aortic T2 (ms)**	**214.3 [204.3**–**226]**	**231.5 [222.8**–**239.3]**	***p*** **= 0.0082**
End Systolic Pressure (mmHg)	110 [102.7–118]	115.3 [110.4–124.2]	*p* = 0.13
PTI Systole (mmHg.s/min)	2307 [2086.7–2520]	2597.2 [2151.8–2833.8]	*p* = 0.24
PTI Diastole (mmHg.s/min) *	3439.3 [3044.7–3758.3]	3168.3 [3051–3353.3]	*p* = 0.63
**Buckberg SEVR (%)**	**148.3 [130**–**168.3]**	**129.5 [119.7**–**145.2]**	***p*** **= 0.040**
**P1 Height (mmHg)**	**29 [24.7**–**31]**	**39.8 [36.2**–**46.3]**	***p*** **< 0.001**
Period (ms)	804.3 [732–955.7]	853 [807.9–991.4]	*p* = 0.47
P2/P1 (%)	133.3 [124–143.3]	139.2 [129.7–143.3]	*p* = 0.70
**Forward Pulse Height (mmHg)**	**26 [23**–**28.3]**	**35.9 [30.2**–**41.4]**	***p*** **< 0.001**
**Reflected Pulse Height (mmHg)**	**16.7 [14.3**–**19]**	**23.7 [21.4**–**26.2]**	***p*** **< 0.001**
Reflection Magnitude (%)	63 [56.7–69]	69.5 [63.1–72.2]	*p* = 0.36

Data are presented as the median [interquartile range] regardless of their distribution. The indices that showed significant differences between Groups I and IV are highlighted. Asterisk marks were added to items which showed significant differences in the pairwise comparisons but were not significant between Groups I and IV. SBP, systolic blood pressure; DBP, diastolic blood pressure; PP, pulse pressure; MP, mean pressure; MAP, mean arterial pressure; AP, augmentation pressure; AIx, augmentation index; PTI, pressure–time index; SEVR, subendocardial viability ratio; vs., versus.

**Table 3:** Echocardiographic parameters: comparison between Group I and Group IV.

	Group I	Group IV	Group I vs. Group IV
	(n = 53)	(n = 18)	
IVSd (mm)	10.2 [8.8–10.8] (n = 44)	9.8 [8.9–10.6] (n = 18)	*p* = 0.96
LVDd (mm)	45.9 [42.8–50.6] (n = 44)	45.9 [42.8–50.4] (n = 18)	*p* = 1.00
LAD (mm) *	37.6 [33.6–40.8] (n = 38)	36.5 [34.6–38.4] (n = 18)	*p* = 1.00
LV-DT (ms)	210 [168–263] (n = 43)	225.5 [206–272.5] (n = 18)	*p* = 0.46
EF (%) *	67 [61.8–73] (n = 44)	68 [65.3–69] (n = 18)	*p* = 0.96
CO (L/min) *	4.4 [3.4–5.5] (n = 43)	4 [2.9–4.9] (n = 18)	*p* = 0.66
E/A	0.9 [0.8–1.2] (n = 38)	0.9 [0.7–1.2] (n = 18)	*p* = 0.89
**e′ (cm/s)**	**6.7 [5.2**–**8] (n = 42)**	**5 [4.1**–**5.8] (n = 15)**	***p*** **= 0.035**
**E/e′**	**9.7 [7.9**–**11.3] (n = 40)**	**13.2 [11.1**–**15.1] (n = 15)**	***p*** **= 0.026**

Data are presented as the median [interquartile range] regardless of their distribution. The indices that showed significant differences between Group I and Group IV are highlighted. Asterisk marks were added to items which showed significant differences in the pairwise comparisons but were not significant between Groups I and IV. Numbers in parentheses indicate the number of patients whose values were obtained. IVSd, diastolic interventricular septal dimension; LVDd, left ventricular end-diastolic dimension; LAD, left atrial diameter; LV-DT, left ventricular deceleration time; EF, ejection fraction; CO, cardiac output; vs., versus.

## Data Availability

The original contributions presented in this study are included in the article; further inquiries can be directed to the corresponding author.

## Abbreviations

The following abbreviations are used in this manuscript:

AVI	Arterial velocity pulse index
API	Arterial pressure–volume index
PWV	Pulse wave velocity
CKD	Chronic kidney disease
BP	Blood pressure
Aix	Augmentation index
SEVR	Buckberg subendocardial viability ratio
EF	Ejection fraction
SD	Standard deviation
ANOVA	Analysis of variance
LDL-C	Low-density lipoprotein cholesterol
HDL-C	High-density lipoprotein cholesterol
BMI	Body mass index
HT	Hypertension
DM	Diabetes mellitus
HL	Hyperlipidemia
CAD	Coronary artery disease
PAD	Peripheral artery disease
CHF	Chronic heart failure
VHD	Valvular heart disease
Af	Atrial fibrillation
BUN	Blood urea nitrogen
eGFR	Estimated glomerular filtration rate
TC	Total cholesterol
TG	Triglyceride
SBP	Systolic blood pressure
DBP	Diastolic blood pressure
PP	Pulse pressure
MP	Mean pressure
MAP	Mean arterial pressure
AP	Augmentation pressure
PTI	Pressure–time index
IVSd	Diastolic interventricular septal diameter
LVDd	Left ventricular end-diastolic dimension
LAD	Left atrial diameter
LV-DT	Left ventricular deceleration time
CO	Cardiac output
